# The effect of cultural and linguistic diversity on the timeliness of prostate cancer treatment: a registry-based retrospective cohort study

**DOI:** 10.1007/s10552-025-02074-4

**Published:** 2025-10-10

**Authors:** Koku Sisay Tamirat, Michael James Leach, Nathan Papa, Jeremy Millar, Eli Ristevski

**Affiliations:** 1 School of Rural Health, Monash University, 15 Sargeant Street, Warragul, VIC 3820 Australia; 2 School of Rural Health, Monash University, Bendigo, VIC Australia; 3 School of Public Health and Preventive Medicine, Monash University, Melbourne, VIC Australia; 4 School of Translational Medicine, Monash University, Melbourne, VIC Australia; 5 Radiation Oncology, Alfred Health, Melbourne, VIC Australia

**Keywords:** Prostate cancer, Definitive treatment delay, Culturally and linguistically diverse backgrounds

## Abstract

**Purpose:**

To examine associations between culturally and linguistically diverse (CALD) status and definitive treatment delay among individuals diagnosed with intermediate- or high-risk prostate cancer (PCa) in Victoria, Australia.

**Methods:**

Data were sourced from the Victorian Prostate Cancer Outcomes Registry (PCOR-Vic). Individuals with index diagnoses of intermediate- or high-risk PCa (February 2009–August 2022) who were receiving PCa-directed definitive treatment (radical prostatectomy or radiotherapy) were included. CALD status was defined as being born in non-English-speaking countries versus mainly English-speaking countries (MESC) or Australia. Additionally, using preferred spoken language, CALD individuals were categorized into English-speaking CALD and non-English-speaking CALD individuals. Binary logistic regression was used to examine associations between CALD status and definitive treatment delay (> 90 days from diagnosis to treatment). Mediation analysis was undertaken using generalized structural equation modeling.

**Results:**

Of 13,625 participants, 2,455 were from CALD backgrounds (18%). Nine per cent of 2,455 CALD individuals preferred speaking languages other than English. Median days to definitive treatment were longer for non-English-speaking CALD (92) compared to Australian-born (64) individuals. Non-English-speaking CALD individuals experienced significantly (adjusted odds ratio = 2.54, 95% confidence interval = 1.92–3.38) greater treatment delay than Australian-born individuals, adjusted for sociodemographic and clinical factors. The association between CALD status and definitive treatment delay was fully mediated by non-English-speaking CALD individuals being diagnosed mostly at public institutions.

**Conclusion:**

Non-English-speaking-CALD individuals experienced greater delays in the definitive treatment of intermediate- and high-risk PCa than Australian-born individuals, which is largely explained by the type of diagnosing health institution. More timely treatment for non-English-speaking-CALD will require less delay in public hospitals.

**Supplementary Information:**

The online version contains supplementary material available at 10.1007/s10552-025-02074-4.

## Introduction

Globally, prostate cancer (PCa) is the second most common cancer diagnosed among men, with 1.4 million new cases in 2020 and this number is expected to double by 2040 [1, 2]. In Australia in 2022, PCa accounted for 27% of all new cancer cases, 13% of cancer deaths in males and 13% of annual health system expenditure on male cancers [3, 4]. PCa outcomes in Australia remain among the best in the world, ranking alongside 25 countries that have PCa survival rates of 90% and above [5]. In Australia, the 5-year relative survival for PCa has increased from 60% for men diagnosed between 1987 and 1991 to 96% for men diagnosed between 2013 and 2017 [4]. However, disparities exist between certain population groups because of who they are, where they live or came from [4]. Individuals from culturally and linguistically diverse (CALD) backgrounds, which can include immigrants and/or individuals who speak languages other than English in Anglophone countries, continue to face challenges in accessing high-quality care and achieving cancer outcomes [6–8]. In Australia, the proportion of the total population born overseas has increased from 23% in 2000 to 31% in 2023; approximately one in five (22%) Australians in 2022 spoke a language other than English at home [9]. Although there is no aggregated age-standardized PCa incidence rate for CALD individuals, an estimate from the Victorian Cancer Registry by region of birth indicates that individuals born overseas generally have a lower mean age-standardized incidence rate compared to Australian-born individuals, except for those born in North America [10]. Nonetheless, there is compelling evidence that CALD individuals in Australia and other high-income countries have had less access to PCa screening [8, 11], presented with advanced stage PCa at diagnosis [12–14], had less access to treatment or received suboptimal treatment [12, 14, 15], and had worse overall survival [16]. Cancer Australia endorsed the Australian Cancer Plan, which puts equity at the center of cancer care and has prioritized certain population groups, including CALD individuals that have been left behind in achieving equitable cancer outcomes [17].

Timely initiation of anti-cancer treatment is a key indicator of healthcare safety and quality, reflecting the availability, accessibility, and coordination of cancer care [18]. As PCa is typically a slow-growing tumor, there is no universally agreed-upon timeframe for optimal timing to commence PCa treatment, and consequently, inconsistent thresholds exist when defining treatment delay [19]. The median interval from PCa diagnosis to treatment initiation varies significantly across different countries, ranging from 79 to 172 days in the United States (US) and Swedish settings, respectively [20, 21] These nuances may be influenced by each country’s health system, clinical practices, and population contexts, affecting logical comparisons between countries. In Australian settings, the median treatment interval from diagnosis to first receipt of definitive treatment ranged from 58 to 65 days [22, 23] with inconsistent cut-offs used to define treatment delay: 30 days in the state of Victoria and 70 days in the state of Queensland. Furthermore, neither of the Australian studies provided data on the timeliness of care among CALD patients diagnosed with PCa; instead, these individuals were excluded from the study due to their limited proficiency in the English language [22].

Evidence from prior studies has shown that a definitive treatment delay (time to surgery or radiation therapy [RT]) of greater than three months in patients with localized (intermediate or high-risk) PCa was associated with poor pathological outcomes, less disease control, and higher rates of postoperative erectile dysfunction and incontinence [24, 25]. Prolonged waiting times to treatment initiation may also induce patient dissatisfaction, fear of progression, and perceptions of unwarranted delays [26, 27]. Some of the factors that may contribute to PCa treatment delay include patients’ race/ethnicity, health insurance, socioeconomic disadvantage, PCa treatment modalities, and health system factors such as diagnosing health institution [22, 24, 28]. Australian and international studies demonstrated that the timeliness of cancer care was significantly shaped by the types of health services, with private healthcare system associated with lower delays [22, 29, 30]. Along with these factors, shared decision-making—aimed at empowering patients with PCa to choose, delay, or avoid aggressive local therapies based on their individual needs, values, and preferences—may also affect the timeliness of treatment [27]. It is also recognized that patient (e.g., culture, language, and ethnicity), provider, and system-level factors can add further layers of complexity to shared decision-making and treatment planning for localized PCa [27, 31, 32].

The ‘Optimal care pathways for men with prostate cancer’ guide the delivery of high-quality, safe, equitable, consistent, and timely PCa care in Australia [33]. This guideline emphasizes the importance of tailoring PCa care to the cultural and linguistic contexts of individuals with PCa [33]. As cultural and linguistic contexts influence individuals’ perceptions of cancer risk, interactions with oncologists, and engagement in preventive and therapeutic health services [8, 27, 34]. The same guideline also suggests an optimal timeline of three months following a confirmed diagnosis to initiate treatment for non-metastatic PCa within Australian settings [33]. The Australian public health system further applies an urgency-based categorisation of surgery, with prostatectomy classified into category 2 (semi-urgent)—procedures clinically indicated within 90 Despite the existence of benchmarks for optimal management of PCa, there are no known studies that have evaluated the timeliness of PCa treatment and examined the relationship between CALD status and definitive treatment delay in patients with PCa in the Australian context.

Our study, therefore, sought to estimate the median time to definitive treatment, disaggregated by CALD status, and to investigate whether delays in definitive treatment for intermediate- and high-risk PCa differ by CALD status in Victoria, Australia. Additionally, the study aimed to identify factors contributing to disparities in definitive treatment delays, defined as delays exceeding 90 days from diagnosis.

## Methods

### Study design and data source

We conducted a retrospective cohort study using data sourced from the Victorian Prostate Cancer Outcomes Registry (PCOR-Vic). PCOR-Vic is a PCa-specific clinical quality registry established in 2009 through a pilot program funded by Cancer Australia and administered by Monash University [36]. This registry is an International Organization for Standardization (ISO-27001)-accredited web-based registry that collects patient, provider, and healthcare system data [36]. Overall, PCOR-Vic was established to monitor, understand, and investigate disparities in the quality of PCa care and treatment outcomes [36, 37].

Patients who may be eligible for PCOR-Vic may be identified through the Victorian Cancer Registry (VCR) (state-based cancer registry) [36]. Every quarter, PCOR-Vic receives VCR extracts for all PCa notifications from contributing health services in the past three months, facilitated by ethics approval with health institutions. PCOR-Vic considers the inclusion of individuals with a confirmed index diagnosis of PCa who are aged 18 years or older [37]. PCOR-Vic recruits participants in an opt-out approach, which maximizes inclusion. Approximately 90 Victorian health services contribute to PCOR-Vic, which captures and reports on approximately 87% of newly diagnosed PCa cases in Australia’s second most populous state: Victoria. The registry provides ongoing information on the clinical presentation, management, and outcomes of patients with PCa [37].

Data items collected by PCOR-Vic are sourced from participants’ medical records within the health services (e.g., diagnostic and treatment data), data collection tools, follow-up interviews, and, through linkages, the VCR and the state’s vital status registry (i.e., Victorian Registry of Births, Deaths, and Marriages) [37]. For example, data on sociodemographic characteristics (e.g., residential address postcodes, preferred spoken language, and country-of-birth) were obtained from notifications while receiving extracts from the VCR [37]. Country-of-birth and language data are routinely recorded in health services*;* however, country-of-birth data may not be entirely accurate and complete (i.e., non-missing) in VCR and—by extension—PCOR-Vic because VCR only receives a subset of registrants’ hospitalization records from notifying health services. Originally, information about the preferred spoken language is collected to translate explanatory statements (in addition to the English language version), promoting inclusivity and reaching non-English-speaking individuals. PCOR-Vic’s participant recruitment and data collection process is detailed elsewhere [36, 38].

### Participants

**Fig. 1:**
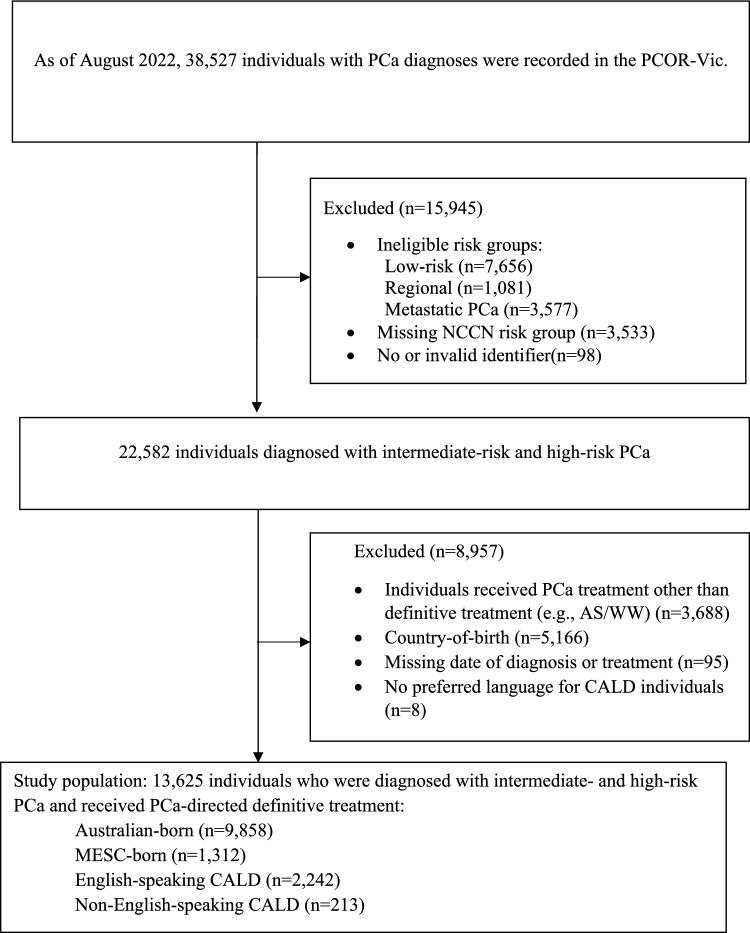
Sample extraction flow diagram. *AS/WW* active surveillance or watchful waiting, *NCCN* National Comprehensive Cancer Network, *PCa* prostate cancer, *PCOR-Vic* Victorian Prostate Cancer Outcomes Registry

We included individuals who were diagnosed with index intermediate- or high-risk PCa between February 2009 and August 2022, and who received PCa-directed definitive treatment (radical prostatectomy [RP] and RT with or without androgen deprivation therapy [ADT]). Subjects were excluded if they had unknown or missing data on country-of-birth, dates of diagnosis and initial treatment, or National Comprehensive Cancer Network (NCCN) risk group. Additionally, any participants with missing identifiers/invalid records or CALD individuals with missing language data were excluded (Fig. 1).

## Measures and definitions

### Time to definitive treatment

Time to definitive treatment (number of days) was defined as the interval from the date of PCa diagnosis to the date of initial definitive treatment commencement. The date of biopsy-confirmed diagnosis was obtained from individuals’ medical records. The date of definitive treatment could include the date of surgery (if RP was the initial treatment received), the date of RT (if the individual was treated with curative-intent RT, that is, external beam RT or brachytherapy), or the date of neoadjuvant ADT (if the individual was treated with neoadjuvant ADT plus RT, whichever came first).

### Definitive treatment delay

The outcome of interest in the present study was a clinically meaningful definitive treatment delay. This was defined as a time from biopsy to definitive treatment of greater than 90 days. For individuals with intermediate- or high-risk PCa who received RP, RT, or any combination of these, the time to definitive treatment was dichotomised into ≤ 90 days (timely initiation), the reference group, and > 90 days (delayed initiation), the non-reference group. This definition aligns with the *‘Optimal Care Pathway for Men with Prostate Cancer*’, which recommends that the optimal time from PCa diagnosis to definitive treatment should not exceed three months. This three-month period is considered a safe delay, allowing sufficient time to facilitate decision-making and treatment planning [33].

### Culturally and linguistically diverse backgrounds

Individuals’ CALD attributes, such as country-of-birth, immigration status, and preferred spoken language, were identified as factors that shape interactions with the health system and utilization of PCa care [39, 40]. Cancer Australia, an agency of the Australian Government, has prioritized individuals from CALD backgrounds to ensure they have the best possible cancer outcomes [17]. In the present study, CALD status is the main exposure variable. As recommended by the Australian Bureau of Statistics (ABS), CALD status can be defined by minimum CALD-defining indicators based on the individual’s: country-of-birth, language other than English spoken at home, English language proficiency, and Indigenous status [41]. Of these indicators, country-of-birth and preferred spoken language are the most commonly documented and reported CALD-defining attributes [40]. Based on the individual’s country-of-birth data and preferred spoken language by individuals born in mainly English-speaking countries, we determined CALD status. The study population was categorized into four groups: 1) immigrants to Australia born in mainly non-English-speaking countries (NESC) whose preferred language was English (i.e., English-speaking CALD individuals), 2) immigrants to Australia born in non-English-speaking countries (NESC) whose preferred language was not English (non-English-speaking CALD individuals), 3) immigrants to Australia from mainly English-speaking countries (MESC) (i.e., MESC-born individuals), and 4) Australian-born individuals (i.e., the reference population). PCOR-Vic collects preferred spoken language (but not English language proficiency or language spoken at home) for follow-up purposes. Individuals’ preferred spoken language was determined using information from medical records on the need for an interpreter and classified as English or non-English.

### Sociodemographic characteristics

We extracted data on the following sociodemographic characteristics from the PCOR-Vic database: age-at-diagnosis (years), year-of-diagnosis, and residential area derived from the Modified Monash Model (MM) classifications of individuals’ residential postcodes (grouped as metropolitan [MM1] and non-metropolitan areas [MM2-7]) [42]. We used MM with the most frequent observations for a single postcode given multiple MM scores. The area-level socioeconomic status (SES) of study participants was determined from their residential postcodes using the Socioeconomic Indexes for Areas—Index of Relative Socioeconomic Disadvantage (SEIFA-IRSD) quintiles [43]. Regions of birth were defined using the Standard Australian Classifications of Countries (SACC) [44]. Health services where individuals seek initial cancer diagnosis and care often shape their treatment journey, care experience, timeliness, and continuity of care [45]. In light of this, the health services where PCa cases were first diagnosed were classified as private and public diagnosing health institutions. In Australia, private health institutions are those where patients require privately paid health insurance to obtain treatment, while public health institutions are those where treatment is covered under the Australian Government’s Medicare scheme [46].

### Clinical characteristics

Clinical data included the NCCN risk group [47], prostate-specific antigen (PSA) value, International Society of Urological Pathology (ISUP) grade group [48], which ranges from low grade (1) to high grade (5), and the type of PCa treatment received within 12 months of diagnosis.

PCa-directed definitive treatments received within 12 months of diagnosis included surgery (i.e., RP), RT, or any combination of these. Treatment with RT means attempted curative treatment with any form of ionizing radiation (essentially external beam RT and/or brachytherapy), without completed RP, but with or without pre-radiation ADT (“neoadjuvant ADT”).

### Statistical analysis

We summarized sociodemographic and clinical data descriptively with medians and interquartile ranges. Univariable and multivariable binary logistic regression models were fitted to examine associations between the CALD background variable (the independent variable of primary interest) and the binary definitive treatment delay variable (the dependent variable). The multivariable model was adjusted sequentially in two steps. The first model included the sociodemographic variables age-at-diagnosis, CALD status, residential area, area-level SES, and year-of-diagnosis, plus two clinical variables: NCCN risk group and definitive treatment modality. The second model included all sociodemographic and clinical variables in the second model, plus the variable for diagnosing health institutions. Both crude odds ratios and adjusted odds ratios (aORs) with 95% confidence intervals (CIs) were used to assess associations between CALD status and definitive treatment delays.

A mediation analysis was undertaken to examine the extent to which diagnosing health institutions and area-level SES explained the associations between CALD status and definitive treatment delays. Mediation analysis is a statistical technique used to explore the mechanisms that explain a potential causal link between the independent and dependent variables using a third intermediary mediator variable. This can help to elucidate, and provide plausible justifications of causal relationship. Two factors, the type of diagnosing health services and area-level SES, were considered intermediate factors within the causal pathway because they have been found to mediate cancer treatment and its timeliness [14, 30]. In a generalized structural equation model (‘gsem’), the Bernoulli family (distribution for binary outcomes) with a logit link function was used for categorical endogenous variables, and the Gaussian family (normal distribution for a continuous variable) with an identity link function was used for the continuous mediator variable [49]. Direct, indirect, and total effects were computed using the post-estimation ‘*nlcom’* command. The model was adjusted for age-at-diagnosis, residential area, year-of-diagnosis, NCCN risk groups, and treatment modalities. The direct, indirect, and total effects were reported based on β coefficients with 95% CIs. A positive coefficient (*β* > 0) that is statistically significant indicates a positive association between exposure, mediator, and outcome variables, whereas a negative coefficient (*β* < 0) that is statistically significant indicates a negative association between exposure, mediator, and outcome variables [50].

For the binary logistic regression and generalized structural equation modeling, we used a complete case approach to handling missing data and default programming in the Stata software. In sensitivity analyses, we performed multiple imputations by chained equations to account for missing data on CALD status and NCCN risk groups. A regression model was refitted with covariate adjustments. We compared the results of our primary analyses (complete case analyses) with those obtained from an imputed dataset in the sensitivity analyses.

A p-value of less than 0.05 was used to declare statistical significance. Data management and statistical analysis were performed using Stata version 16 software (StataCorp LLC, College Station, TX, USA).

## Ethics approval

We used deidentified, individual-level data requested from and supplied by PCOR-Vic. PCOR-Vic obtained informed consent from all participants through an explanatory statement written in both English and translation. PCOR-Vic often provides translated versions of the explanatory statement for individuals whose preferred language was not English or who had a documented need for an interpreter. In PCOR-Vic, the most commonly mentioned languages other than English include Greek, Mandarin, Italian, Vietnamese, Cantonese, Macedonian, Ukrainian, Serbian, Spanish, Russian, Turkish, Arabic, and Polish. The current study received ethical approval from the Monash University Human Research Ethics Committee (project number: 36187).

## Results

### Study population characteristics

Overall, 13,625 individuals with intermediate- or high-risk PCa were analyzed (Table 1). The total population comprised 9,858 Australian-born individuals (72%), 2,242 English-speaking CALD individuals (16%), 1,312 MESC-born individuals (10%), and 213 non-English-speaking CALD individuals (2%). More than three-quarters (77%) of MESC-born individuals were from North-West Europe, whereas CALD individuals (both English-speaking CALD and non-English-speaking CALD) were mainly from South and Eastern Europe (43–45%). Compared to Australian-born individuals, those non-English-speaking CALD individuals were older at diagnosis (median [IQR] age: 67 [61–72] vs 71 [66–75] years). Individuals from CALD backgrounds (both English-speaking and non-English-speaking) were more frequently found to reside in metropolitan areas (87–97% vs 60%) and received a PCa diagnosis through public health services (42–93%). English-speaking CALD (34%) and non-English-speaking CALD (48%) individuals presented with high-risk PCa more frequently than Australian-born individuals (32%). More individuals from CALD backgrounds, particularly those non-English-speaking CALDs with intermediate- or high-risk PCa, than Australian-born individuals received RT (51% vs 26%), and 77% of 109 non-English-speaking CALD individuals received RT with neoadjuvant ADT. Conversely, CALD individuals, particularly non-English-speaking CALDs, received RP less often than Australian-born individuals (49% vs 74%) (Table 1).

**Table 1:** Sociodemographic and clinical characteristics of individuals disaggregated by culturally and linguistically diverse background status, Victoria, Australia (*N* = 13,625) ** Cell size < 5

Characteristics	Frequency, *n* (column %) ^¥^				
	Australian-born (*n* = 9,858)	MESC-born (*n* = 1,312)	CALD-English speaking (*n* = 2,242)	CALD-non-English speaking (*n* = 213)	Overall (*n* = 13,625)
Age at PCa diagnosis, median (IQR)	67 (61–72)	68 (62–73)	68 (64–73)	71 (66–75)	67 (62–72)
Age category					
< 60	2,037 (20.7)	217 (16.5)	317 (14.1)	20 (9.4)	2,591 (19.0)
60–64	1,917 (19.4)	243 (18.5)	374 (16.7)	28 (13.1)	2,562 (18.8)
65–69	2,590 (26.3)	350 (26.7)	632 (28.2)	54 (25.4)	3,626 (26.6)
70–74	1,999 (20.3)	309 (23.6)	536 (23.9)	55 (25.8)	2,899 (21.3)
75 +	1,315 (13.3)	193 (14.7)	383 (17.1)	56 (26.3)	1,947 (14.3)
Regions of birth					
Australia	9,858 (100.0)	–	–	–	9,858 (72.3)
Oceania exc. Australia	–	159 (12.1)	87 (3.9)	–	246 (1.8)
North-West Europe	–	1,015 (77.4)	369 (16.5)	–	1,384 (10.2)
South and Eastern Europe	–	–	969 (43.2)	97 (45.5)	1,066 (7.8)
South-East Asia	–	–	215 (9.6)	41 (19.2)	256 (1.9)
South and Central Asia	–	–	208 (9.3)	**	210 (1.5)
North Africa and the Middle East	–	–	167 (7.4)	21 (9.8)	188 (1.4)
Sub-Saharan Africa	–	77 (5.9)	77 (3.4)	**	157 (1.1)
North-East Asia	–	–	99 (4.4)	43 (20.2)	142 (1.0)
Americas	–	61 (4.6)	51 (2.3)	6 (2.8)	118 (0.9)
Residential area					
Metropolitan [MM-1]	5,891 (59.8)	956 (72.9)	1,948 (86.9)	207 (97.2)	9.002 (66.1)
Non-metropolitan [MM 2-7]	3,967 (40.2)	356 (27.1)	294 (13.1)	6 (2.8)	4,623 (33.9)
SEIFA-IRSD quintiles					
Lower	1,128 (11.4)	95 (7.2)	259 (11.5)	44 (20.7)	1,526 (11.2)
Lower-middle	1,589 (16.1)	160 (12.2)	249 (11.1)	29 (13.6)	2,027 (14.9)
Middle	1,538 (15.6)	207 (15.8)	398 (17.8)	51 (23.9)	2,194 (16.1)
Middle-upper	2,197 (22.3)	329 (25.1)	525 (23.4)	47 (22.1)	3,098 (22.7)
Upper	3,406 (34.6)	521 (39.7)	811 (36.2)	42 (19.7)	4,780 (35.1)
Diagnosing health institution					
Private	6,670 (67.7)	852 (64.9)	1,301 (58.0)	14 (6.6)	8,837 (64.9)
Public	3,188 (32.3)	460 (35.1)	941 (42.0)	199 (93.4)	4,788 (35.1)
Year of diagnosis					
2009–2016	3,115 (31.6)	394 (30.0)	802 (35.8)	40 (18.8)	4,351 (31.9)
2017–2019	4,142 (42.0)	554 (42.2)	885 (39.5)	88 (41.3)	5,669 (41.6)
2020–2022	2,601 (26.4)	364 (27.7)	555 (24.7)	85 (39.9)	3,605 (26.5)
NCCN risk group					
Intermediate-risk	6,739 (68.4)	925 (70.5)	1,471 (65.6)	110 (51.6)	9,245 (67.8)
High-risk	3,119 (31.6)	387 (29.5)	771 (34.4)	103 (48.4)	4,380 (32.2)
Diagnostic PSA (ng/mL)					
< 10	7,235 (74.1)	930 (71.6)	1,545 (69.8)	131 (62.4)	9,841 (73.0)
10–20	1,887 (19.3)	271 (20.9)	517 (23.3)	56 (26.7)	2,731 (20.2)
> 20	644 (6.6)	97 (7.5)	152 (6.9)	23 (10.9)	916 (6.8)
Not recorded^§^	92	14	28	**	137
Diagnostic Gleason score					
ISUP 1 (GS ≤ 6)	189 (1.9)	28 (2.1)	52 (2.3)	5 (2.4)	274 (2.0)
ISUP 2 (GS 3 + 4)	4,674 (47.5)	612 (46.7)	979 (43.7)	82 (38.7)	6,347 (46.6)
ISUP 3 (GS 4 + 3)	2,455 (24.9)	358 (27.3)	581 (25.9)	45 (21.2)	3,439 (25.3)
ISUP 4 (GS 4 + 4; 5 + 3; 3 + 5)	1,204 (12.2)	172 (13.1)	315 (14.1)	37 (17.4)	1,728 (12.7)
ISUP 5 (GS 4 + 5; 5 + 4; 5 + 5)	1,328 (13.5)	142 (10.8)	314 (14.0)	43 (20.2)	1,827 (13.4)
Not recorded^§^	8	–	**	**	10
Treatment modality					
Radical prostatectomy	7,256 (73.6)	961 (73.3)	1,582 (70.6)	104 (48.8)	9,903 (72.7)
Radiation therapy	2,602 (26.4)	351 (26.7)	660 (29.4)	109 (51.2)	3,722 (27.3)
Radiation therapy modality	(*n* = 2,602)	(*n* = 351)	(*n* = 660)	(*n* = 109)	(*n* = 3,721)
Radiation therapy alone	1,021 (39.2)	145 (41.3)	233 (35.3)	25 (22.9)	1,423 (38.3)
Radiation with ADT	1,581 (60.8)	206 (58.7)	427 (64.7)	84 (77.1)	2,298 (61.7)

Preferred non−English spoken languages include Greek, Mandarin, Italian, Vietnamese, Cantonese, Macedonian, Ukrainian, Serbian, Spanish, Russian, Turkish, Arabic, Polish, and other aboriginal English languages. CALD: defined as being born in mainly non−English-speaking countries. CALD−English speaking refers to CALD individuals whose preferred language is English. CALD−Non−English speaking: refers to CALD individuals born whose preferred language is not English

*ADT* Androgen deprivation therapy, *CALD* Culturally and Linguistically Diverse Backgrounds, *GS* Gleason score, *ISUP* International Society of Urology Pathology, *IQR* Interquartile Range, *NCCN* National Comprehensive Cancer Network, MESC−Mainly English−Speaking Countries, *MM* Modified Monash Model, *PSA* Prostate−specific antigen, *PCa* Prostate cancer, *SEIFA−IRSD* Socioeconomic Index for Area Index of Relative Socioeconomic Disadvantage

<5 (NS: not stated) describe cells having a small frequency

^¥^column percentage calculated unless otherwise specified (age reported as median)

^§^Not recorded frequencies for diagnostic PSA, Gleason score, radiation therapy modality, and types of surgical procedure were excluded from the denominator for percentage calculation

### Time from prostate cancer diagnosis to definitive treatment

**Fig. 2:**
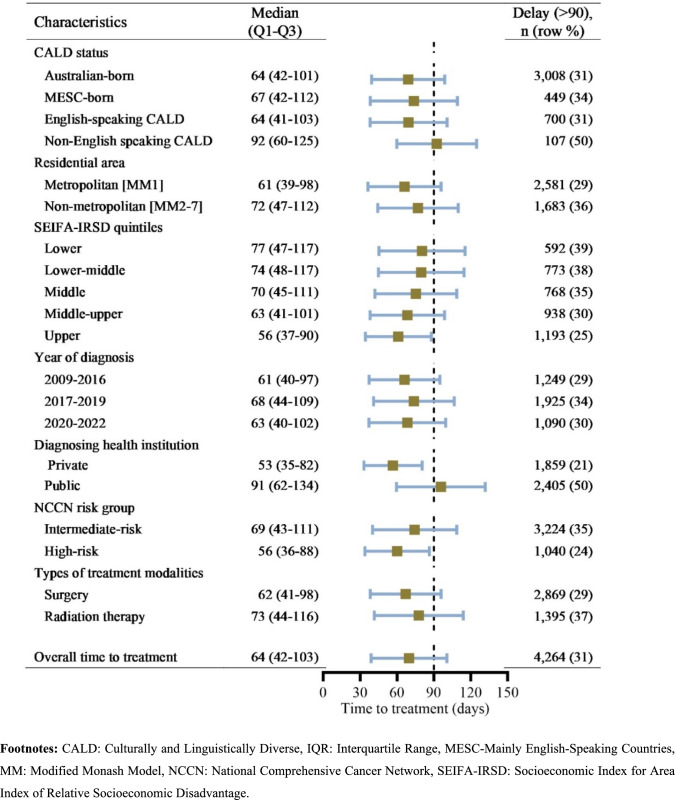
Median (IQR: Q1–Q3) time to definitive treatment and proportions of delay for individuals from culturally and linguistically diverse backgrounds. *CALD* Culturally and Linguistically Diverse, *IQR* Interquartile Range, *MESC* Mainly English-Speaking Countries, *MM* Modified Monash Model, *NCCN* National Comprehensive Cancer Network, *SEIFA-IRSD* Socioeconomic Index for Area Index of Relative Socioeconomic Disadvantage

There were clinically relevant differences in the time from PCa diagnosis to definitive treatment by CALD status, with MESC-born and non-English CALD individuals experiencing a longer median time to initiation of definitive treatment than Australian-born individuals (67 days for MESC-born and 92 days for non-English-speaking CALD individuals) (Fig. 2). For the overall population, the median time to commence definitive treatment meaningfully differed by area-level SEIFA-IRSD quintiles and health system where PCa was first diagnosed (Fig. 2 and supplementary Table 1).

Across the whole sample, 4,264 of 13,625 individuals (31%) had a definitive treatment delay greater than 90 days, with the proportion considerably higher for non-English-speaking CALD individuals (50%) compared to MESC-born individuals (34%) and Australian-born individuals (31%). The proportions of delay for English-speaking CALD individuals (31%) were comparable (Fig. 2 and Supplementary Table 1).

### Associations between culturally and linguistically diverse background status and definitive treatment delay

In the multivariable logistic regression analysis, adjusting for clinical and sociodemographic variables (model 1), MESC-born individuals and CALD-English-speaking individuals had 28% (aOR = 1.28, 95% CI = 1.13–1.45) and 16% (aOR = 1.16, 95% CI = 1.05–1.29) higher odds of experiencing definitive treatment delay compared to Australian-born individuals, respectively. Similarly, the odds of definitive treatment delays were 2.54-fold greater for CALD individuals whose preferred language was not English (non-English-speaking CALD) compared to Australian-born individuals (aOR = 2.54, 95% CI = 1.92–3.38) (supplementary Table 2 provides OR estimates of both adjusted models).

**Fig. 3:**
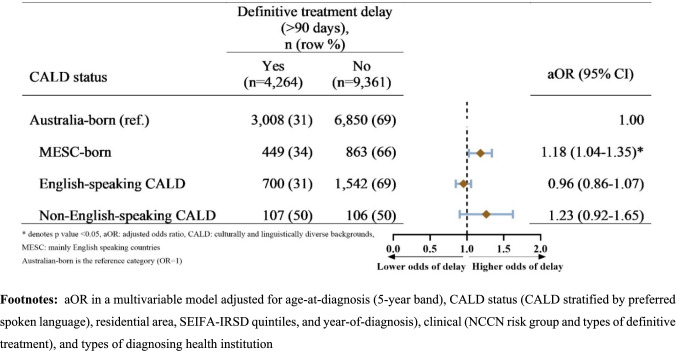
Forest plots showing the association between men’s culturally and linguistically diverse backgrounds status and delay in definitive treatment. aOR in a multivariable model adjusted for age-at-diagnosis (5-year band), CALD status (CALD stratified by preferred spoken language), residential area, SEIFA-IRSD quintiles, and year-of-diagnosis), clinical (NCCN risk group and types of definitive treatment), and types of diagnosing health institution

In a maximally adjusted multivariable binary logistic regression analysis, which controlled for health institution diagnosis alongside sociodemographic and clinical factors (model 2), MESC-born individuals had 18% (aOR = 1.18, 95% CI = 1.04–1.35) higher odds of experiencing definitive treatment delay compared to Australian-born individuals. However, the associations between delays in definitive treatment and English-speaking CALD and non-English-speaking CALD attenuated, and the confidence interval crossed one, with an effect estimate change of over 10% for CALD (aOR = 0.96, 95% CI = 0.86–1.07) and CALD-non-English-speaking (aOR = 1.23, 95% CI = 0.92–1.65) individuals (Fig. 3).

Further stratified data analysis by diagnosing health institutions with adjustment for previously listed covariates showed that: (1) there was no significant excess odds of definitive treatment delay associated with CALD status in both strata; (2) the odds of definitive treatment delay among non-English-speaking CALD individuals were similar across those diagnosed in private (aOR = 1.10, 95% CI: 0.29–4.16) and public (aOR = 1.17, 95% CI: 0.86–1.60) health services, and (3) changes in effect estimates exceeded by greater than 10% from the unstratified (crude) estimate (cOR = 2.30, 95% CI = 1.75–3.02). Meanwhile, compared with their Australian-born counterparts, MESC-born individuals diagnosed in public health services had 24% higher odds of experiencing definitive treatment delay (aOR = 1.24, 95% CI = 1.01–1.52), while MESC-born individuals diagnosed in private institutions had no greater odds of definitive treatment delay yet a similar effect estimate (aOR = 1.12, 95% CI = 0.94–1.34) (Supplementary Table 3).

In a mediation analysis, health institutions where PCa was first diagnosed fully mediated and area-level SES partially mediated, except for MESC-born individuals, the associations between CALD status and definitive treatment delay. The indirect effect (β = 5.205, 95% CI = 4.397–6.012; *p* < 0.001) and total effects estimates (β = 5.450, 95% CI = 4.621–6.288; *p* < 0.001) suggest that non-English-speaking CALD individuals were associated with greater delays in definitive treatment through disproportional diagnosis in the public health service. Similarly, SEIFA-IRSD has been shown to partially mediate the association between CALD-non-English-speaking individuals and delay in definitive treatment (*β*_indirect effect_ = 0.057, 95% CI: 0.025–0.089; *p* < 0.001) (Supplementary Table 4). The directed acyclic graph (Supplementary Fig. 1) provides a specific estimate for exposure (CALD-non-English speaking), potential mediators (SEIFA quintile and type of diagnosing health institution), and outcome (delays in definitive treatment) pathways.

### Sensitivity analysis result

On sensitivity analysis, we found comparable results; using multiple imputations by chained equations did not materially alter the overall findings of our study. No substantial change in the adjusted odds ratio (maximally adjusted model) for the association between definitive treatment delay and CALD status was found [MESC (aOR = 1.18, 95% CI = 1.04–1.34)]. However, there was a 17% relative change in the aOR estimate for the association between non-English-speaking CALD delays in definitive treatment, with the confidence interval now excluding one in the sensitivity analysis (aOR = 1.48, 95% CI = 1.10–2.00) (Supplementary File 1).

## Discussion

Our retrospective cohort study, conducted in Victoria, Australia, found that 31% of individuals with PCa experienced substantial delays in definitive treatment, exceeding 90 days. Non-English-speaking CALD individuals experienced more frequent delays in definitive treatment compared to Australian-born individuals after controlling for demographic, socioeconomic, and clinical factors. However, when further adjustment was made to the type of diagnosing health institution (private or public), the association was attenuated for CALD individuals. This finding indicates that differences based on the types of health services where PCa was first diagnosed have fully mediated and elucidated disparities in delays for definitive treatment by CALD status. Furthermore, SES (SEIFA quintile) also partially mediated the association between CALD status (for both English-speaking CALD and non-English-speaking CALD) and delays in definitive treatment. Our results underscore that healthcare planners and researchers evaluating delays in PCa treatment should consider contributing factors such as health service and SES factors among CALD individuals.

Our findings of delays in definitive treatment among CALD individuals with PCa align with previous research results. A retrospective cohort study in the US found that the time from diagnosis to definitive treatment was statistically significantly longer for ethnically/racially minoritized and non-English-speaking groups compared to their counterparts, after adjusting for sociodemographic, clinical, and type of health insurance [28]. A growing body of evidence shows that individuals from CALD backgrounds face multifaceted challenges in accessing healthcare [6, 51]. For instance, Australian studies among individuals born in Anglo-Australian and NESC backgrounds indicated that those from CALD backgrounds are unfamiliar with local healthcare services, prefer traditional healing methods, and experience language and communication difficulties, which in turn influences health service uptake [34, 52].

Our analyses also provide noteworthy insights into how SES and the types of health institutions involved in diagnostic workup mediate the effect of CALD on definitive treatment delays among individuals with intermediate- and high-risk PCa. Notably, the total effects of non-English-speaking CALD on delays in definitive treatment were largely explained by differences in the PCa diagnostic workup at private health services based on CALD status. This aligns with the theoretical perspective, highlighting that the effects of ethnicity on disparities of worse PCa outcomes among ethnic minorities were largely due to the indirect effects of racism on socioeconomic disadvantage and poor access to healthcare, not merely due to intrinsic variations [53]. Similarly, our study’s findings show that disparities in delays of PCa definitive treatment — beyond the optimal timeframe — were driven by the indirect effect of CALD status on SES disadvantage and less investigation in private health services. This is particularly important for the Australian Cancer Plan, which advocates for equitable and enhanced consumer experience among general cancer patients. The plan proposed a person-centered, integrated navigation approach, co-designed and tailored to meet men’s cultural and social needs, ensuring the right support at the right time throughout the cancer continuum [17].

It is well understood that the private healthcare sector offers faster diagnostic and treatment pathways and is an option for patients who are able to self-fund their care. Our analysis reinforces this: private health services were associated with considerably shorter median diagnosis to treatment intervals (53 days vs 91 days) and lower definitive treatment delays (21% vs 50%) compared to the public system. Previous Australian and international research that included individuals with localized PCa and lung cancer showed that healthcare in private systems was associated with shorter treatment intervals, supporting our findings [22, 28, 29]. Furthermore, disparities in access to private health services also explained differences in time to cancer surgery based on ethnicity [30]. However, there is a growing concern that expanding the private health sector may weaken health system planning and integration, as private institutions operate autonomously and may be less inclined to cooperate [54]. Moreover, experts argue that private health services are often criticized for providing more low-value care [55, 56].

In contrast, delays that fall short of the standards of care are widespread in the public healthcare system. Meanwhile, Australia’s Medicare system ensures that all Australians have access to a range of free-of-charge or subsidized healthcare services [57]. However, the system is strained due to long waiting lists and backlogs resulting from high demand for care [46]. To mitigate this, the Australian National Elective Surgery Urgency Categorisation assigned prostatectomy to a category 2 level of urgency for publicly funded hospitals [35]; however, a significant portion of patients from the public system still experience delays. These findings indicate the need for auditing and monitoring PCa care pathways, which are necessary to standardize care and minimize differences in PCa treatment timeliness [36].

Another important consideration is the role of SES disadvantage (as measured by SEIFA quintile) in contributing to differences in definitive treatment delays by CALD status. Our analyses revealed a clear SES gradient in definitive treatment delays, with individuals in the lower SEIFA quintile (most disadvantaged) (39%) experiencing delays more frequently compared to those in the upper SEIFA quintile (25%). A greater percentage of non-English-speaking CALD individuals were from the lower SEIFA areas (most disadvantaged) compared to Australian-born individuals (11% vs 21%). This result was in line with prior Australian data indicating that CALD individuals with cancer face significantly greater SES disadvantages, including a lower annual income of ≥ $70,000 (23% vs 29%) and private health insurance coverage (49% vs 62%), as well as being from lower SEIFA areas [58]. This underscores how intersectionality is playing a role in introducing delays in the definitive treatment of PCa among individuals from CALD backgrounds. Enhanced consumer experience is one of the six strategic objectives acknowledged and prioritized by the Australian Cancer Plan [17, 59].

Our findings of disparities in the timeliness of definitive treatment and contributory factors (SES and the diagnosing health institution) may be ‘the tip of the iceberg’ regarding the challenges of non-English-speaking CALD individuals in accessing timely cancer care. Although there is no universally agreed-upon timeframe for optimal timing to commence PCa treatment, delays in PCa definitive treatment raise concerns; prolonged delay for PCa treatment is associated with biochemical recurrence, histopathological upgrading, and poor functional outcomes [24, 25, 60]. Delays in definitive treatment in this study may have important contributions in providing insights to predict patient outcomes, as prior evidence highlights the association of biochemical recurrence and poor pathological outcomes with delays of 6 to 9 Given these negative effects of treatment delay on patient outcomes, the health system should address treatment delays and contributing factors among CALD individuals with localized PCa. Beyond this, our findings of delayed definitive PCa treatment suggest a need for further work to foster public–private partnerships, promote culturally safe and linguistically appropriate patient education about PCa, promote treatment decision support, and deliver optimal, equitable care to the population from CALD backgrounds as well as those born in MESC and Australia.

The present study has several strengths. Our study is the first Australian analysis of the timeliness of PCa treatment by CALD status, examining the association between CALD status and the delay in definitive PCa treatment. By using the date of confirmed diagnosis, including the biopsy date, to calculate treatment intervals, we potentially minimized overestimations that would have been associated with the PSA testing date. The study also has several limitations. Although the proportion of individuals in the study population born overseas was comparable with the estimated proportion of Victorians born overseas (32% and 28%, respectively) [61], the operational definition of CALD status using an individual’s country-of-birth only could have led to the misclassification of second- or higher-generation immigrants. Our study could have been confounded by unmeasured factors, including years lived in Australia or level of acculturation, comorbidities, provider characteristics, and treatment decision-making-related factors that have been shown to impact healthcare experiences and contribute to treatment delays. In particular, this study has no data on the PCa treatment decision-making and planning process. We attempted to mitigate this issue by adjusting for treatment modality in regression models; however, treatment modality is an imperfect marker of the treatment decision-making and planning process. It is understood that PCa management is subject to a preference-sensitive approach, including communication and shared decision-making between patients and the care team, which may create delays in PCa treatment [27]. In the same way, the length of time a person has lived in Australia is an important factor influencing utilization of PCa care [8], but it is not recorded in PCOR-Vic. Our approach of subgrouping CALD individuals into English-speaking CALD individuals and non-English-speaking CALD individuals can provide insights into the level of acculturation and migration pathways, partially overcoming this limitation.

Though demographic attributes in the PCOR-Vic registry resembled those of first-generation Australian immigrants, 22% of cases recorded in the PCR-Vic registry had missing country-of-birth data. This suggests that the results of our study may have been influenced by selection bias. Such bias may not be problematic because, firstly, missing country-of-birth data are likely to be largely missing at random due to VCR and—by extension—PCOR-Vic sourcing these data from a subset of hospitalization records and, secondly, our sensitivity analysis revealed that missing data did not alter the overall significance of our study findings. Due to the large sample size, detecting a difference of 3–4 days, for instance, between MESC-born and Australian-born men may be trivial or have little to no clinical implications.

## Conclusions

We found that delays in definitive treatment for intermediate- and high-risk PCa varied by CALD status, largely due to the diagnosing health institution (public or private). Individuals born outside of Australia, particularly non-English-speaking CALD individuals, disproportionately experienced delays in receiving definitive treatment for localized PCa, attributed to SES and investigation at public health services. The contributing factors identified in this study help guide healthcare professionals and cancer control authorities in their efforts to foster equitable and timely management of PCa.

## Supplementary Information

Below is the link to the electronic supplementary material.

Supplementary file1 (DOCX 16 KB)

Supplementary file2 (DOCX 95 KB)

## Data Availability

Data from the Australian Bureau of Statistics and the Australian Government Department of Health and Aged Care are publicly available on their respective websites. Other data analyzed are not publicly available, but applications can be made to the PCOR-Vic steering committee for data access.
