# Supplementary material for: The effect of cultural and linguistic diversity on the timeliness of prostate cancer treatment: a registry-based retrospective cohort study
Source: Cancer Causes Control. 2025 Oct 10;36(12):1975–88. doi: 10.1007/s10552-025-02074-4 (PMC12630315; doi:10.1007/s10552-025-02074-4)
Supplement: Supplementary file 1 — Supplementary file1 (DOCX 16 KB) [file 10552_2025_2074_MOESM1_ESM.docx]

Supplementary TableS1: Sensitivity analysis examining the association between culturally and linguistically diverse background status and definitive treatment delay among intermediate- and high-risk prostate cancer patients (n=18,008)

| Variables | Crude OR (95%CI) | Adjusted OR (95%) |
| --- | --- | --- |
| CALD status |  |  |
| Australia-born | 1.00 | 1.00 |
| MESC-born | 1.15 (1.02-1.29)* | 1.18 (1.04-1.34)* |
| English-speaking CALD | 1.03 (0.93-1.13) | 1.05 (0.93-1.15) |
| Non-English speaking CALD | 1.88 (1.42-2.50)* | 1.48 (1.10-2.00)* |

Footnotes: *denotes statistically significant at a 0.05 P-value

The multivariable model (adjusted OR) was adjusted for age-at-diagnosis (5-year band), residential area, SEIFA-IRSD quintiles, and year-of-diagnosis, clinical (NCCN risk group and types of definitive treatment), and types of diagnosing health institution

Supplementary TableS2: Sensitivity analysis stratified by diagnosing health services to examine the association between culturally and linguistically diverse background status and definitive treatment delay among intermediate- and high-risk prostate cancer patients (n=18,008)

| Variables | Public health services (n=7,913) | | | Private health services (n=10,095) | |
| --- | --- | --- | --- | --- | --- |
|  | Crude OR (95%CI) | | Adjusted OR (95%CI) | Crude OR (95%CI) | Adjusted OR (95%) |
| CALD status |  | |  |  |  |
| Australia-born | 1.00 | | 1.00 | 1.00 | 1.00 |
| MESC-born | 1.24 (1.03-1.49) | | 1.30 (1.08-1.57)* | 1.04 (0.88-1.23) | 1.06 (0.88-1.27) |
| English-speaking CALD | | 0.97 (0.85-1.09) | 1.05 (0.92-1.20) | 0.95 (0.81-1.12) | 0.99 (0.84-1.17) |
| Non-English speaking CALD | | 1.39 (1.04-1.87) | 1.57 (1.15-2.14)* | 1.10 (0.45-2.70) | 1.09 (0.42-2.83) |

Footnotes: *denotes a statistically significant at a 0.05 P-value

The multivariable model (aOR) was adjusted for age-at-diagnosis (5-year band), residential area, SEIFA-IRSD quintiles, and year-of-diagnosis, and clinical (NCCN risk group and types of definitive treatment)
