# Supplementary material for: The effect of cultural and linguistic diversity on the timeliness of prostate cancer treatment: a registry-based retrospective cohort study
Source: Cancer Causes Control. 2025 Oct 10;36(12):1975–88. doi: 10.1007/s10552-025-02074-4 (PMC12630315; doi:10.1007/s10552-025-02074-4)
Supplement: Supplementary file 2 — Supplementary file2 (DOCX 95 KB) [file 10552_2025_2074_MOESM2_ESM.docx]

Supplementary Table 1: Median (IQR) time to definitive treatment and n (%) of patients substantial treatment delay (>90 days) overall and by sociodemographic and clinical characteristics, culturally and linguistically diverse backgrounds status, Victoria, Australia (N=13,625). ** Cell size < 5

| Characteristics | Australian-born (n=9,858) | | MESC-born (n=1,312) | | English-speaking CALD (n=2,242) | | Non-English-speaking CALD (n=213) | | Overall (n=13,625) | |
| --- | --- | --- | --- | --- | --- | --- | --- | --- | --- | --- |
|  | Median (IQR) | Delay (>90), n (row %) | Median (IQR) | Delay (>90), n (row %) | Median (IQR) | Delay (>90), n (row %) | Median (IQR) | Delay (>90), n (row %) | Median (IQR) | Delay (>90), n (row %) |
| Overall | 64 (42-101) | 3,008 (31%) | 64(41-103) | 449 (34%) | 66 (42-105) | 700 (31%) | 92 (60-125) | 107 (50) | 64 (42-103) | 4,264 (31%) |
| Age at diagnosis (grouped) |  |  |  |  |  |  |  |  |  |  |
| <60 | 64 (42-105) | 640 (31) | 72 (47-117) | 72 (33) | 65 (43-106) | 98 (31) | 76 (56-124) | 9 (45) | 65 (42-107) | 819 (32) |
| 60-64 | 66 (42-104) | 620 (32) | 69 (42-112) | 84 (35) | 66 (42-112) | 136 (36) | 124 (78-187) | 18 (64) | 67 (42-106) | 858 (33) |
| 65-69 | 66 (43-105) | 844 (33) | 63 (40-114) | 120 (34) | 64 (41-102) | 202 (32) | 101 (64-141) | 32 (59) | 66 (42-105) | 1,198 (33) |
| 70-74 | 61 (41-97) | 561 (28) | 71 (47-109) | 118 (38) | 65 (42-101) | 167 (31) | 93 (64-118) | 29 (53) | 63 (42-101) | 875 (30) |
| 75+ | 58 (36-92) | 343 (26) | 58 (36-101) | 55 (29) | 60 (35-91) | 97 (25) | 68 (46-105) | 19 (34) | 58 (36-93) | 514 (26) |
| Regions of birth |  |  |  |  |  |  |  |  |  |  |
| Australia | 64 (42-101) | 3,008 (31) | -- |  | -- | -- | -- | -- | 64 (42-101) | 3,008 (31) |
| Oceania exc. Australia | -- | -- | 69 (44-110) | 55 (35) | 70 (47-107) | 28 (32) | -- | -- | 69 (45-109) | 83 (34) |
| North-West Europe | -- | -- | 67 (42-111) | 342 (34) | 67 (44-106) | 124 (33) | -- | -- | 67 (43-111) | 466 (34) |
| South and Eastern Europe | -- | -- | -- | -- | 62 (41-100) | 292 (30) | 78 (56-122) | 44 (45) | 64 (42-102) | 336 (32) |
| South-East Asia | -- | -- | -- | -- | 63 (36-105) | 70 (33) | 103 (70-152) | 28 (68) | 68 (40-111) | 98 (38) |
| South and Central Asia | -- | -- | -- | -- | 69 (43-110) | 72 (35) | 94 (64-123) | ** | 69 (43-110) | 73 (35) |
| North Africa and the Middle East | -- | -- | -- | -- | 56 (35-96) | 44 (26) | 100 (72-123) | 12 (57) | 61 (39-101) | 56 (30) |
| Sub-Saharan Africa | -- | -- | 64 (39-113) | 30 (39) | 73 (43-113) | 28 (36) | 55 (14-76) | ** | 69 (39-113) | 58 (37) |
| North-East Asia | -- | -- | -- | -- | 62 (33-86) | 22 (22) | 83 (56-123) | 19 (44) | 65 (42-98) | 41 (29) |
| Americas | -- | -- | 70 (45-112) | 22 (36) | 81 (49-118) | 20 (39) | 94 (83-120) | ** | 77 (49-118) | 45 (38) |
| Residential area |  |  |  |  |  |  |  |  |  |  |
| Metropolitan [MM-1] | 60 (39-98) | 1,582 (27) | 64 (42-112) | 309 (32) | 63 (40-101) | 586 (30) | 92 (60-123) | ** | 61 (39-98) | 2,581 (29) |
| Non-metropolitan [MM 2-7] | 72 (46-115) | 1,426 (36) | 77 (49-117) | 140 (39) | 76 (49-112) | 114 (39) | 93 (57-134) | ** | 72 (47-112) | 1,683 (36) |
| SEIFA-IRSD quintiles |  |  |  |  |  |  |  |  |  |  |
| Lower | 78 (47-117) | 445 (39) | 82 (57-129) | 44 (46) | 72 (47-106) | 86 (33) | 77 (52-107) | 17 (39) | 77 (47-117) | 592 (39) |
| Lower-middle | 73 (47-118) | 604 (38) | 78 (52-126) | 62 (39) | 72 (47-107) | 91 (37) | 94 (66-126) | 16 (55) | 74 (48-117) | 773 (38) |
| Middle | 69 (45-108) | 510 (33) | 76 (47-127) | 84 (41) | 69 (46-113) | 145 (36) | 97 (64-127) | 29 (57) | 70 (45-111) | 768 (35) |
| Middle-upper | 61 (40-97) | 609 (28) | 70 (43-111) | 119 (36) | 69 (46-112) | 185 (35) | 96 (56-123) | 25 (53) | 63 (41-101) | 938 (30) |
| Upper | 56 (37-90) | 840 (25) | 56 (37-95) | 140 (27) | 54 (35-88) | 193 (24) | 84 (62-148) | 20 (48) | 56 (37-90) | 1,193 (25) |
| Diagnosing health institution |  |  |  |  |  |  |  |  |  |  |
| Private | 53 (36-82) | 1,407 (21) | 54 (36-86) | 194 (23) | 50 (32-79) | 255 (20) | 52 (14-80) | ** | 53 (35-82) | 1,859 (21) |
| Public | 91 (62-134) | 1,601 (50) | 101 (64-148) | 255 (55) | 87 (61-126) | 445 (47) | 94 (62-125) | 104 (52) | 91 (62-134) | 2,405 (50) |
| Year of diagnosis |  |  |  |  |  |  |  |  |  |  |
| 2009-2016 | 64 (41-104) | 870 (28) | 63 (40-107) | 128 (32) | 61 (31-97) | 231 (29) | 91 (64-147) | 20 (50) | 61 (40-97) | 1,249 (29) |
| 2017-2019 | 68 (44-109) | 1,385 (33) | 71 (45-113) | 208 (38) | 67 (43-111) | 294 (33) | 81 (59-112) | 38 (43) | 68 (44-109) | 1,925 (34) |
| 2020-2022 | 62 (39-101) | 753 (29) | 64 (42-109) | 113 (31) | 65 (40-104) | 175 (32) | 98 (57-134) | 49 (58) | 63 (40-102) | 1,090 (30) |
| NCCN risk group |  |  |  |  |  |  |  |  |  |  |
| Intermediate-risk | 68 (43-109) | 2,269 (34) | 70 (44-115) | 352 (38) | 69 (45-111) | 531 (36) | 104 (71-147) | 72 (65) | 69 (43-111) | 3,224 (35) |
| High-risk | 56 (36-88) | 739 (24) | 58 (39-91) | 97 (25) | 55 (34-85) | 169 (22) | 72 (50-109) | 35 (34) | 56 (36-88) | 1,040 (24) |
| Diagnostic PSA (ng/mL) |  |  |  |  |  |  |  |  |  |  |
| <10 | 64 (42-102) | 2,221 (31) | 67 (43-112) | 327 (35) | 64 (41-103) | 485 (31) | 82 (63-123) | 63 (48) | 64 (42-104) | 3,096 (31) |
| 10-20 | 64 (42-104) | 599 (32) | 67 (41-115) | 96 (35) | 69 (43-100) | 163 (32) | 104 (70-131) | 34 (61) | 66 (42-105) | 892 (33) |
| >20 | 56 (36-90) | 156 (24) | 67 (42-85) | 23 (24) | 54 (32-103) | 43 (28) | 56 (48-116) | 9 (39) | 57 (36-91) | 231 (25) |
| Not recorded | 69 (44-98) | 32 (35) | 70 (49-85) | ** | 62 (47-105) | 9 (32) | 83 (57-103) | ** | 69 (46-98) | 45 (33) |
| Diagnostic Gleason score |  |  |  |  |  |  |  |  |  |  |
| ISUP 1 (GS ≤6) | 108 (59-195) | 109 (58) | 131 (76-201) | 20 (71) | 131 (59-204) | 34 (65) | 251 (218-317) | 5 (100) | 113 (62-204) | 168 (61) |
| ISUP 2 (GS 3+4) | 70 (45-113) | 1,670 (36) | 74 (45-118) | 244 (40) | 73 (45-117) | 378 (39) | 103 (71-143) | 52 (63) | 71 (45-115) | 2,344 (37) |
| ISUP 3 (GS 4+3) | 61 (40-95) | 672 (27) | 65 (42-105) | 117 (33) | 63 (42-99) | 170 (29) | 103 (70-127) | 29 (64) | 62 (41-98) | 988 (29) |
| ISUP 4 (GS 4+4; 5+3; 3+5) | 58 (39-88) | 287 (24) | 60 (42-94) | 43 (25) | 52 (31-76) | 68 (22) | 65 (49-83) | 9 (24) | 58 (39-88) | 407 (24) |
| ISUP 5 (GS 4+5; 5+4; 5+5) | 50 (34-81) | 269 (20) | 50 (30-77) | 25 (18) | 52 (31-76) | 50 (16) | 69 (48-104) | 12 (28) | 51 (33-80) | 356 (19) |
| Not recorded | 46 (25-62) | ** | -- | -- | -- | -- | - |  | 39 (14-57) | ** |
| Treatment modality |  |  |  |  |  |  |  |  |  |  |
| Radical prostatectomy | 61 (41-97) | 2,028 (28) | 64 (41-106) | 308 (32) | 62 (40-99) | 471 (30) | 106 (71-144) | 62 (60) | 62 (41-98) | 2,869 (29) |
| Radiation therapy | 73 (44-118) | 980 (38) | 76 (47-118) | 141 (40) | 70 (47-113) | 229 (35) | 72 (51-107) | 45 (41) | 73 (44-116) | 1,395 (37) |
| Radiation therapy modality |  |  |  |  |  |  |  |  |  |  |
| Radiation therapy alone | 106 (74-160) | 636 (62) | 112 (82-159) | 100 (69) | 112 (79-166) | 150 (64) | 120 (100-155) | 22 (88) | 110 (76-161) | 908 (64) |
| Radiation with ADT | 55 (34-85) | 344 (22) | 55 (38-82) | 41 (20) | 57 (35-82) | 79 (19) | 63 (47-94) | 23 (27) | 56 (35-84) | 487 (21) |
| **^Footnotes:^** ^ADT: Androgen deprivation therapy, CALD: Culturally and Linguistically Diverse Backgrounds, CT: Computed Tomography, GS: Gleason score, ISUP: International Society of Urology Pathology, IQR: Interquartile Range, NCCN: National Comprehensive Cancer Network, MESC-Mainly English-Speaking Countries, MM: Modified Monash Model, MRI: Magnetic Resonance Imaging, PSA: Prostate-specific antigen, PCa: Prostate cancer, PET: Positron Emission Tomography, RARP: Robot-assisted radical prostatectomy, SEIFA-IRSD: Socioeconomic Index for Area Index of Relative Socioeconomic Disadvantage. Preferred non-English spoken languages include Greek, Mandarin, Italian, Vietnamese, Cantonese, Macedonian, Ukrainian, Serbian, Spanish, Russian, Turkish, Arabic, Polish, and other aboriginal English languages, and others^  ^Australian-born is the reference population.^  ^CALD‡‡: defined as being born in non-English speaking countries^  ^CALD: defined as being born in mainly non-English speaking countries. CALD-English speaking refers to CALD individuals whose preferred language is English. CALD-Non-English speaking: refers to CALD individuals born whose preferred language is not English^ | | | | | | | | | | |

Supplementary Table 2: Univariable and multivariable logistic regression analyses examining the associations between culturally and linguistically diverse background status (and disaggregated by preferred spoken language) and definitive treatment delay among individuals diagnosed with intermediate- and high-risk prostate cancer, Victoria, Australia (n=13,625)

| Variables | Definitive treatment delay  (>90 days), n (row %) | | | Univariable model  cOR (95%CI) | Multivariable models, aOR (95%CI) | | |
| --- | --- | --- | --- | --- | --- | --- | --- |
|  | Yes  (n=4,264) | | No  (n=9,367) |  | Model 1‡ | | Model 2‡‡ |
| Age at diagnosis in years (median, IQR) | 67 (62-72) | 68 (62-73) | | 0.96 (0.93-0.98) | 0.91 (0.89-0.94) | 0.97 (0.94-1.00) | |
| CALD status |  |  | |  |  |  | |
| Australia-born (ref.) | 3,008 (31) | 6,850 (69) | | 1.00 | 1.00 | 1.00 | |
| MESC-born | 449 (34) | 863 (66) | | 1.18 (1.05-1.34) | 1.28 (1.13-1.45) | 1.18 (1.04-1.35) | |
| English-speaking CALD | 700 (31) | 1,542 (69) | | 1.03 (0.94-1.14) | 1.16 (1.05-1.29) | 0.96 (0.86-1.07) | |
| Non-English-speaking CALD | 107 (50) | 106 (50) | | 2.30 (1.75-3.02) | 2.54 (1.92-3.38) | 1.23 (0.92-1.65) | |
| Residential area |  |  | |  |  |  | |
| Metropolitan [MM1] | 2,581 (29) | 6,421 (71) | | 1.00 | 1.00 | 1.00 | |
| Non-metropolitan [MM2-7] | 1,683 (36) | 2,940 (64) | | 1.42 (1.32-1.54) | 1.27 (1.16-1.39) | 1.10 (1.00-1.20) | |
| SEIFA-IRSD quintiles |  |  | |  |  |  | |
| Lower | 592 (39) | 934 (61) | | 1.00 | 1.00 | 1.00 | |
| Lower-middle | 773 (38) | 1,254 (62) | | 0.97 (0.85-1.11) | 0.96 (0.84-1.11) | 1.00 (0.87-1.16) | |
| Middle | 768 (35) | 1,426 (65) | | 0.85 (0.74-0.97) | 0.84 (0.73-0.97) | 0.91 (0.79-1.06) | |
| Middle-upper | 938 (30) | 2,160 (70) | | 0.69 (0.60-0.78) | 0.74 (0.65-0.85) | 0.89 (0.78-1.03) | |
| Upper | 1,193 (25) | 3,587 (75) | | 0.52 (0.46-0.59) | 0.58 (0.51-0.66) | 0.82 (0.71-0.94) | |
| Year of diagnosis |  |  | |  |  |  | |
| 2009-2016 | 1,249 (29) | 3,102 (71) | | 1.00 | 1.00 | 1.00 | |
| 2017-2019 | 1,925 (34) | 3,744 (66) | | 1.28 (1.17-1.39) | 1.23 (1.13-1.34) | 1.22 (1.12-1.34) | |
| 2020-2022 | 1,090 (30) | 2,515 (70) | | 1.08 (0.98-1.19) | 1.01 (0.92-1.12) | 1.00 (0.90-1.11) | |
| NCCN risk group |  |  | |  |  |  | |
| Intermediate-risk | 3,224 (35) | 6,021 (65) | | 1.00 | 1.00 | 1.00 | |
| High-risk | 1,040 (24) | 3,340 (76) | | 0.58 (0.54-0.63) | 0.52 (0.48-0.57) | 0.43 (0.40-0.48) | |
| Types of treatment modalities |  |  | |  |  |  | |
| Surgery | 2,869 (29) | 7,034 (71) | | 1.00 | 1.00 | 1.00 | |
| Radiation therapy | 1,395 (37) | 2,327 (63) | | 1.47 (1.36-1.59) | 1.75 (1.60-1.92) | 1.28(1.16-1.41) | |
| Diagnosing health institution | | |  |  |  |  | |
| Public | 2,405 (50) | 2,383 (50) | | 1.00 | -- | 1.00 | |
| Private | 1,859 (21) | 6,978 (79) | | 0.26 (0.24-0.28) | -- | 0.26 (0.24-0.28) | |
| **^Footnotes:^** ^aOR: Adjusted Odds Ratio, CALD: Culturally and Linguistically Diverse, cOR: Crude Odds Ratio, SEIFA-IRSD: Socioeconomic Index for Area—Index of Relative Socioeconomic Disadvantage. MESC: Mainly English-Speaking Countries, NCCN: National Comprehensive Cancer Network. Preferred non-English spoken languages include Greek, Mandarin, Italian, Vietnamese, Cantonese, Macedonian, Ukrainian, Serbian, Spanish, Russian, Turkish, Arabic, Polish, and other aboriginal English languages, and others^  ^Australian-born is the reference population.^  ^CALD‡‡: defined as being born in non-English speaking countries^  ^CALD: defined as being born in mainly non-English speaking countries. CALD-English speaking refers to CALD individuals whose preferred language is English. CALD-Non-English speaking: refers to CALD individuals born whose preferred language is not English^  ^Step-by-step adjustments of covariates in the multivariable models:^  ^Model 1‡: Contains sociodemographic (age-at-diagnosis (5-year band), CALD status (CALD divided by preferred language), residential area, SEIFA-IRSD quintiles, and year-of-diagnosis and clinical variables (NCCN risk group, types of definitive treatment)^  ^Model 2‡‡: Contains sociodemographic (age-at-diagnosis (5-year band), CALD status (CALD stratified by preferred language), residential area, SEIFA-IRSD quintiles, and year-of-diagnosis), clinical (NCCN risk group and types of definitive treatment), and types of diagnosing health institution^ | | | | | | | |

Supplementary Table 3: Stratified analysis examining the association between culturally and linguistically diverse status (stratified by spoken language) and definitive treatment delay types of diagnosing health institution where PCa was first diagnosed among individuals with intermediate- and high-risk prostate cancer in Victoria, Australia (N=13,625)

| CALD status | Unstratified estimate,  cOR (95%CI) | Stratifications by types of diagnosing health institution | | | |
| --- | --- | --- | --- | --- | --- |
|  |  | Private (n=8,837) | | Public (n=4,788) | |
|  |  | cOR (95%CI) | aOR^¥^ (95%CI) | cOR (95%CI) | aOR^¥^ (95%CI) |
| Australian-born (ref.) | 1.00 | 1.00 | 1.00 | 1.00 | 1.00 |
| MESC-born | 1.18 (1.05-1.34) | 1.10 (0.93-1.30) | 1.12 (0.94-1.34) | 1.23 (1.01-1.50) | 1.24 (1.01-1.52) |
| English-speaking CALD | 1.03 (0.94-1.14) | 0.91 (0.79-1.06) | 0.99 (0.85-1.16) | 0.89 (0.78-1.03) | 0.90 (0.76-1.05) |
| Non-English speaking CALD | 2.30 (1.75-3.02) | 1.02 (0.28-3.66) | 1.10 (0.29-4.16) | 1.09 (0.81-1.45) | 1.17 (0.86-1.60) |
| ^Footnotes: aOR: Adjusted Odds Ratio, CALD‡‡: Culturally and Linguistically Diverse cOR: Crude Odds Ratio, SEIFA-IRSD: Socioeconomic Index for Area—Index of Relative Socioeconomic Disadvantage. MESC: Mainly English-Speaking Countries, NCCN: National Comprehensive Cancer Network. preferred non-English spoken language includes Greek, Mandarin, Italian, Vietnamese, Cantonese, Macedonian, Ukrainian, Serbian, Spanish, Russian, Turkish, Arabic, Polish, and other aboriginal English Language, and others^  ^Australian-born is the reference population.^  ^aOR¥: for stratified analysis, the multivariable model was adjusted for age-at-diagnosis (5-year band), residential area, SEIFA-IRSD quintiles, year-of-diagnosis, NCCN risk group, and types of definitive treatment^  ^CALD: defined as being born in mainly non-English speaking countries. CALD-English speaking refers to CALD individuals whose preferred language is English. CALD-Non-English speaking: refers to CALD individuals born whose preferred language is not English^ | | | | | |

Supplementary Table 4: Direct, indirect, total effects of culturally and linguistically diverse background status (stratified by preferred spoken language) on definitive treatment delay among individuals diagnosed intermediate- and high-risk prostate cancer in Victoria, Australia (n=13,625)

| CALD status | Effect | β coefficient 95%CI | P-value | Proportion mediated |
| --- | --- | --- | --- | --- |
| MESC-born | Indirect effect through SES | -0.004 (-0.009 – 0.0002) | 0.063 | NS |
|  | Indirect effect through diagnosing health institution | 0.375 (0.205-0.546) | <0.001 |  |
|  | Total indirect effect | 0.371 (0.200-0.541) | <0.001 | 69% |
|  | Direct effect | 0.167 (0.037-0.298) | 0.012 |  |
|  | Total effect | 0.538 (0.324-0.752) | <0.001 |  |
| English-speaking CALD | Indirect effect through SES | 0.017 (0.007-0.028) | 0.001 | 2% |
|  | Indirect effect through diagnosing health institution | 1.008 (0.859-1.157) | <0.001 | 100% |
|  | Total indirect effect | 1.028 (0.879-1.177) | <0.001 |  |
|  | Direct effect | -0.048 (-0.158-0.061) | 0.391 |  |
|  | Total effect | 0.980 (0.798-1.160) | <0.001 |  |
| Non-English CALD | Indirect effect through SES | 0.057 (0.025-0.089) | <0.001 | 1% |
|  | Indirect effect through diagnosing health institution | 5.205 (4.397-6.012) | <0.001 | 95% |
|  | Total indirect effect | 5.262 (4.455-6.068) | <0.001 |  |
|  | Direct effect | 0.199 (-0.091-0.489) | 0.179 |  |
|  | Total effect | 5.450 (4.621-6.288) | <0.001 |  |
| ^Footnotes: The mediation model was adjusted to age-at-diagnosis (5-year band), residential area, year-of-diagnosis, NCCN risk groups, and treatment modality. CALD: Culturally and Linguistically Diverse, CI: Confidence Intervals, MESC: Mainly English-Speaking Countries, NCCN: Nationally Comprehensive Cancer Network^  ^Australian-born is the reference population.^  ^CALD‡‡: defined as being born in non-English speaking countries.^  ^Statistically significant indirect effect estimates coefficients >0 mean that CALD indirectly contributed to definitive treatment delay through potential mediators (socioeconomic disadvantage and less access to private diagnosing health institutions).^  ^Statistically significant total effect estimates coefficients (which include both direct and indirect effects) >0 means CALD leads to increased definitive treatment delay in either mechanism (directly or indirectly).^ | | | | |

| 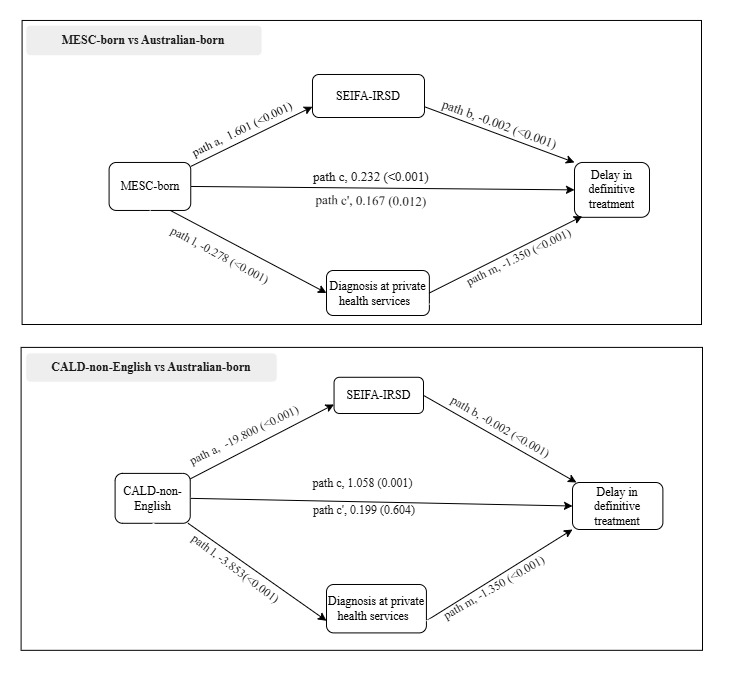**Footnotes:** Abbreviations: CALD: culturally and linguistically diverse backgrounds, MESC-born: mainly English-speaking countries, SEIFA-IRSD: Socioeconomic Index for Area- Index of relative socioeconomic disadvantage.  **For CALD-non-English vs Australian-born:** Path (CALD-non-English to SEIFA-quintile) demonstrates that non-English speaking CALD is significantly associated with lower SEIFA quintiles. Path b (SEIFA to delay in definitive treatment) indicates that as the SEIFA-IRSD quintile increases, delays in definitive significantly decrease. Path l (CALD-non-English to diagnosis at private diagnosis) demonstrates that non-English speaking CALD individuals are less often diagnosed at private health services. Path m (diagnosis at private health service to delay in definitive treatment) indicates that PCa diagnosis at the private health service is associated with a lower probability of delays in definitive treatment. Path c (CALD-non-English to delay in definitive) demonstrates a direct effect estimate (unadjusted for mediators) that non-English speaking CALD status is positively and directly associated with delays in definitive treatment. Path c’ demonstrates that when mediators were considered in the model, the association between non-English speaking CALD status and delay in definitive treatment becomes statistically insignificant (supporting the assumptions of mediation). |
| --- |

Supplementary Figure 1: the directed acyclic graph (DAG) depicting the association between CALD-non-English-speaking individuals and delays in definitive treatment through potential mediators.
